# A randomized multi-center phase II trial of the angiogenesis inhibitor Cilengitide (EMD 121974) and gemcitabine compared with gemcitabine alone in advanced unresectable pancreatic cancer

**DOI:** 10.1186/1471-2407-6-285

**Published:** 2006-12-11

**Authors:** Helmut Friess, Jan M Langrehr, Helmut Oettle, Jochen Raedle, Marco Niedergethmann, Christian Dittrich, Dieter K Hossfeld, Herbert Stöger, Bart Neyns, Peter Herzog, Pascal Piedbois, Frank Dobrowolski, Werner Scheithauer, Robert Hawkins, Frieder Katz, Peter Balcke, Jan Vermorken, Simon van Belle, Neville Davidson, Albert Abad Esteve, Daniel Castellano, Jörg Kleeff, Adrien A Tempia-Caliera, Andreas Kovar, Johannes Nippgen

**Affiliations:** 1 Department of General Surgery, University of Heidelberg, Heidelberg, Germany; 2 Department of General and Transplantation Surgery, Charité, Virchow-Clinic, Berlin, Germany; 3 Department of Hematology and Oncology, Charité, Virchow-Clinic, Berlin, Germany; 4 Department of Medicine II, University of Frankfurt Hospital, Frankfurt am Main, Germany; 5 Department of Surgery, University Hospital Mannheim, Mannheim, Germany; 6 Ludwig Blotzmann-Institute for Applied Cancer Research (LBI-ACR Vienna), Kaiser Franz Josef-Spital, Vienna, Austria; 7 Department of Oncology/Hematology, University Hospital Hamburg-Eppendorf, Hamburg, Germany; 8 Department of Internal Medicine, Division of Oncology, Medical University of Graz, Graz, Austria; 9 Department of Medical Oncology, Oncology Center, Vrije Universiteit Brussel, Brussels, Belgium; 10 Department of Gastroenterology, Reinhardt Nieter Krankenhaus, Wilhelmshaven, Germany; 11 Department of Medical Oncology, Henri Mondor Hospital, Creteil Cedex, France; 12 Department of Visceral, Thoracic and Vascular Surgery, University Hospital Carl Gustav Carus, Technical University of Dresden, Dreseden, Germany; 13 Department of Internal Medicine I, Division of Clinical Oncology, University Hospital of Vienna, Vienna, Austria; 14 Department of Medical Oncology, Paterson Institute for Cancer Research, University of Manchester, Manchester, UK; 15 Department of Medicine V, Clinic Darmstadt, Darmstadt, Germany; 16 Department of Medicine, Clinic of the City of St. Poelten, St. Pölten, Austria; 17 Department of Oncology, University Hospital Antwerpen, Edegem, Belgium; 18 Department of Oncology, University Hospital Gent, Gent, Belgium; 19 Department of Medicine, St John's Hospital, Chelmsford, Essex, UK; 20 Department of Oncology, Hospital Germans Trias i Pujol, Badalona, Spain; 21 Department of Oncology, Hospital Universitario 12 de Octubre, Madrid, Spain; 22 Department of Pharmacology and Pharmacokinetics, Merck KGaA, Darmstadt, Germany; 23 Medical Center of Excellence Oncology, Merck KGaA, Darmstadt, Germany

## Abstract

**Background:**

Anti-angiogenic treatment is believed to have at least cystostatic effects in highly vascularized tumours like pancreatic cancer. In this study, the treatment effects of the angiogenesis inhibitor Cilengitide and gemcitabine were compared with gemcitabine alone in patients with advanced unresectable pancreatic cancer.

**Methods:**

A multi-national, open-label, controlled, randomized, parallel-group, phase II pilot study was conducted in 20 centers in 7 countries. Cilengitide was administered at 600 mg/m^2 ^twice weekly for 4 weeks per cycle and gemcitabine at 1000 mg/m^2 ^for 3 weeks followed by a week of rest per cycle. The planned treatment period was 6 four-week cycles. The primary endpoint of the study was overall survival and the secondary endpoints were progression-free survival (PFS), response rate, quality of life (QoL), effects on biological markers of disease (CA 19.9) and angiogenesis (vascular endothelial growth factor and basic fibroblast growth factor), and safety. An ancillary study investigated the pharmacokinetics of both drugs in a subset of patients.

**Results:**

Eighty-nine patients were randomized. The median overall survival was 6.7 months for Cilengitide and gemcitabine and 7.7 months for gemcitabine alone. The median PFS times were 3.6 months and 3.8 months, respectively. The overall response rates were 17% and 14%, and the tumor growth control rates were 54% and 56%, respectively. Changes in the levels of CA 19.9 went in line with the clinical course of the disease, but no apparent relationships were seen with the biological markers of angiogenesis. QoL and safety evaluations were comparable between treatment groups. Pharmacokinetic studies showed no influence of gemcitabine on the pharmacokinetic parameters of Cilengitide and vice versa.

**Conclusion:**

There were no clinically important differences observed regarding efficacy, safety and QoL between the groups. The observations lay in the range of other clinical studies in this setting. The combination regimen was well tolerated with no adverse effects on the safety, tolerability and pharmacokinetics of either agent.

## Background

Cilengitide (EMD 121974, Cyclo-l-Arg-l-Asp-d-Phe-N (Me) l-Val) is a new low-molecular weight anti-angiogenesis agent. It is a cyclic peptide inhibitor of the endothelial cell surface receptors, integrins ανβ 3 and ανβ 5 [1]. Integrins are responsible for cell adhesion to the extracellular matrix. They bind to a number of extracellular matrix components including fibronectin, laminin, collagen, vitronectin, fibrinogen and thrombospondin. These molecules form the vascular matrix and subendothelial basement membrane of blood vessels [2]. The endothelial expression of the extracellular matrix molecules changes during angiogenesis under the regulation of integrins. The key role integrins play in tumor angiogenesis is illustrated by studies showing their involvement in cell migration, proliferation and survival [3]. The integrin ανβ 3, which binds to a number of extracellular matrix proteins, is not generally expressed in normal tissues but is significantly upregulated on blood vessels undergoing angiogenesis [4]. The distinct but functionally related ανβ 5 receptor differs from ανβ 3 in a way that ανβ 3 plays a critical role in basic fibroblast growth factor (bFGF)-stimulated angiogenesis and ανβ 5 in vascular endothelial growth factor (VEGF)-induced angiogenesis [5]. Both ανβ 3 and ανβ 5 are expressed on tumor cells [6-9], and in some cancers expression has been linked with a poor prognosis [10].

Cilengitide inhibits the binding of vitronectin to ανβ 3 with a 50% inhibitory concentration (IC_50_) of 1 nM and to ανβ 5 with an IC_50 _of 140 nM [11]. Cilengitide inhibits tumor-mediated angiogenesis and the growth of human xenografted tumors [12,13]. Non-growth-inhibitory doses of Cilengitide were shown to increase the efficacy of radioimmunotherapy in a breast cancer xenograft model [14]. In vitro studies have shown that Cilengitide not only inhibits angiogenesis but also is directly toxic to ανβ 3- and ανβ 5-expressing tumor cell lines [15]. Phase I data showed that, in patients with advanced solid tumors, Cilengitide (30 mg/m^2 ^to 1600 mg/m^2^) given twice weekly was well tolerated and resulted in prolonged stable disease in 3 patients [16].

Carcinoma of the pancreas is an aggressive human cancer noted for its early metastatic potential and poor prognosis [17]. The disease accounts for around 32,000 deaths a year in the United States [18], and around 47.000 in Western Europe [19]. The median survival time for patients with locally advanced disease is 6 to 10 months and for those with metastases the median survival time is only 3 to 6 months. Most patients present with advanced disease and the symptoms of the disease cause substantial morbidity. It has been suggested that, in addition to the traditional goals of clinical trials (response rate, survival), subjective measures such as disease stabilization and symptom palliation are particularly important for patients with pancreatic cancer [20]. In recent years gemcitabine has replaced 5-fluorouracil-based chemotherapy as the preferred choice for first line palliative chemotherapy in patients with advanced pancreatic cancer. Although it has shown clinical benefit, the median survival time of patients treated with gemcitabine alone is 5.7 months [21] and with gemcitabine in combination with 5-fluoruracil 6.7 months [22]. There is a clear need for effective new agents or adjuncts to existing therapies for patients with cancer of the pancreas.

Pancreatic carcinoma like other tumors also depends on the development of an adequate blood supply through angiogenesis. The pivotal role of angiogenesis in primary tumor growth and metastasis has been recognized many years before. Inhibition of neo-angiogenesis is a new and attractive target for tumor therapy, since it theoretically offers the hope of long-term control of tumor progression. Antiangiogenic therapy offers a number of potential benefits including lack of resistance to some agents, synergistic interaction to other modalities and potentially reduced toxicity compared with conventional agents. Administration of angiogenesis inhibitors might keep the tumor and its metastases stable in growth and let co-administration of cytotoxic drugs kill it. The anti-neoplastic actions of angiogenic inhibitors and cytotoxic agents are clearly different. Treatment with antiangiogenic agents could interact in a positive way with a variety of anti-cancer therapies, and the anti-metastatic and anti-tumor effects of combination therapy were stronger than those of angiogenic inhibitors alone and cytotoxic agents alone.

Towards this goal, the present study was designed to compare the effects of treatment with Cilengitide and gemcitabine with gemcitabine alone. The primary objective of the study was to investigate overall survival in patients with advanced unresectable pancreatic cancer. The secondary objectives were to determine the effects of these therapies on progression-free survival (PFS), response rates, tumor growth control, the biological responses of surrogate tumor markers and angiogenic growth factors, safety and tolerability, and quality of life (QoL). An ancillary study investigated the pharmacokinetics of Cilengitide and gemcitabine in a subset of patients.

## Methods

### Patients

Patients with advanced unresectable pancreatic cancer were enrolled. Eligibility criteria included age ≥ 18 years, diagnosed (histologically or cytologically) with or without metastases, at least one bidimensionally measurable lesion, a Karnofsky performance score of ≥ 70%, a life expectancy of at least 12 weeks, and written informed consent. The main exclusion criteria were prior chemotherapy and/or major surgery related to pancreatic cancer before study entry with the exception of palliative surgery, brain metastases, a pancreatic tumor of neuroendocrine origin, second primary malignancies, inadequate liver and renal functions, inadequate bone marrow reserve, a history of cerebrovascular accident or repeated transient ischemic attacks and cardiac or cardiovascular abnormality. The study was approved by the independent Ethics Committees and by the Local Authorities of the different institutions according to country specific laws before the start of the study.

### Study design and treatment

This was a multi-national, open-label, controlled, randomized, parallel-group, phase II pilot study, conducted in 20 centers in 7 countries (Austria, Belgium, France, Germany, Spain, Switzerland, and the United Kingdom). Eligible patients were randomized to receive either Cilengitide and gemcitabine or gemcitabine alone. The Pocock minimization method [23] was applied for randomization using factors for center and for locally advanced or metastatic disease following the definition according to the tumor-node-metastasis (TNM) classification and histopathological grading system of the International Union Against Cancer. Cilengitide (600 mg/m^2^) was administered twice weekly as a 1 hour intravenous (IV) infusion for 4 consecutive weeks (1 cycle). Gemcitabine (1000 mg/m^2^) was administered as a weekly 30-minute IV infusion once weekly for 3 weeks followed by 1 week of rest (1 cycle). In the drug combination group, on the days on which both drugs were given, Cilengitide was given immediately prior to gemcitabine. The planned treatment period was 24 weeks (6 cycles), but patients responding to either regimen could continue treatment up to a maximum of 48 weeks. Patients experiencing dose-limiting toxicities, as assessed by the National Cancer Institute Common Toxicity Criteria (NCI-CTC) grading criteria, had their treatment reduced, delayed or discontinued. Any change was based on patient hematological and/or non-hematological parameters on the intended day of treatment as well as the maximum drug-induced toxicity grade observed between dosing.

### Survival and tumor response evaluations

Overall survival was defined as the time from the start of study drug administration to death. PFS was defined as the time interval between the date of randomization and the date of disease progression or death whichever came first. If neither was observed the patient was censored at the date of the last follow-up examination. A computed tomography or magnetic resonance imaging scan of target lesions was performed at baseline, weeks 9 and 17, and at the end of treatment to assess tumor size and treatment response. Unscheduled scans were conducted to confirm disease progression. Tumor response was assessed according to the amended World Health Organization (WHO) criteria for response based on the size of target lesions.

### Quality of Life evaluations

Quality of life (QoL) was assessed using the European Organization for Research and Treatment of Cancer (EORTC) QoL core questionnaire (QLQ-C30, Version 3.0) in conjunction with the EORTC Pancreatic Module (EORTC QLQ-PAN26). QLQ-C30 is a self-administered 30-item questionnaire and incorporates 5 functional scales, 3 symptom scales and a global health/QoL scale. EORTC-PAN26 is a 26-item questionnaire addressing pancreas cancer specific symptoms and the side effects from conventional therapy. Assessments were made at weeks 1, 9, 17, 25 and at the end of treatment. QoL data were analyzed descriptively due to the small number of patients enrolled in the study. The descriptive data were interpreted as suggested by Osaba et al. [24] with a mean change of 10 to 20 representing a "moderate" change and a change of greater than 20 representing a "large" change in QoL.

### Biological response

Biological response was assessed by measuring the levels of tumor markers associated with pancreatic cancer (CA 19-9) or angiogenesis (VEGF, bFGF). Measurements by means of commercially available ELISA kits were made of CA 19-9 in serum, VEGF in plasma and serum and bFGF in plasma, serum and urine at weeks 1, 5, 9, 13, 17, 21 and at the final visit.

### Safety

The before treatment and 4-weekly evaluations included a physical examination and laboratory investigations (blood counts, biochemical profile, urinalysis). Assessments were also made of Karnofsky performance status and vital signs. Before the start of the study, at the end of the study and when clinically indicated during the study chest X-rays and 12-lead electrocardiograms were performed. Adverse events were monitored and evaluated for frequency, duration, severity (mild, moderate, severe, life-threatening) and possible relationship to study medication. Toxicity was assessed according to NCI-CTC Version 2. All adverse events whether reported by a patient or observed by an investigator or study nurse were documented, regardless of whether they were considered to be drug-related, together with any medical intervention.

### Pharmacokinetic analysis

An ancillary pharmacokinetic study was carried out using previously described methodology [25,26] in a subset of patients receiving either Cilengitide and gemcitabine or gemcitabine alone. Blood samples were taken following drug administration and the plasma extracted immediately and stored. Blood samples for determination of Cilengitide were collected at the following time points: day 1 immediately before Cilengitide infusion and after 1 h (end of infusion); day 8 immediately before Cilengitide infusion and after 1 h (end of infusion) and 0.5 h, 1 h, 2 h, 3 h, 4 h, 7 h, and 11 h after end of Cilengitide infusion. Blood samples for gemcitabine analysis were collected in both treatment arms on day 1 and day 8 at the following time points: 0 h (immediately before start of infusion of gemcitabine), 0.5 h (at the end of infusion of gemcitabine) and 0.5 h, 1.5 h, 2.5 h, and 3.5 h after end of gemcitabine infusion.

Analysis was carried out at a single laboratory using high-performance liquid chromatography-tandem mass spectrometry. Calculation of pharmacokinetic parameters was performed according to non-compartmental standard methods. Pharmacokinetic analyses included maximum plasma concentration (C_max_), time to maximum concentration (t_max_), area under the curve from time zero to the last sampling time (AUC_0-t_), area under the curve from time zero to infinity (AUC_0-∝_), apparent elimination rate constant (λ_z_), apparent terminal elimination half-life associated with the negative terminal slope (t_1/2_), total body clearance of drug from plasma (CL) and the volume of distribution at steady state (V_ss_). The lower limits of quantification for Cilengitide and gemcitabine in plasma were 1 ng/mL and 50 ng/mL, respectively. For the plasma quality control samples of Cilengitide the interbatch coefficient of variation (CV) was 3.4% to 9.2% and the interbatch accuracy was 97% to 104%. For the plasma quality control samples of gemcitabine, the interbatch CV was 5.6% to 9.4% and the interbatch accuracy was 93.5% to 104%.

### Statistical analysis

The sample size of the trial was determined by clinical feasibility. No inferential statistical comparison of the two treatment arms was planned. The aim was to obtain information on safety and feasibility as well as trends in efficacy by determination of overall survival as primary and progression-free survival (PFS), response rate, quality of life (QoL), effects on biological markers of disease (CA 19.9) and angiogenesis (vascular endothelial growth factor and basic fibroblast growth factor) and safety as secondary endpoints. Confidence intervals were presented at the 95%-confidence level. The data of this phase II pilot- and feasibility study, are presented in a descriptive manner. The primary analysis of survival (time to death from any cause) was conducted using the intention-to-treat (ITT) population. An additional supportive analysis using the Per Protocol (PP) population was conducted. The secondary objectives were to determine PFS, response rates, safety and tolerability, QoL, biological response, and pharmacokinetics. Time to death or progression was analyzed using PROC LIFETEST.

## Results

### Patient characteristics

Eighty-nine patients were enrolled, randomized and included in the ITT population. Three patients died before receipt of the study drug making 86 patients evaluable for safety. Seventy-six patients were included in the PP population. Of the 13 major protocol violations, 12 received less than 4 weeks of treatment and one met an exclusion criterion. The demographic and clinical characteristics of the ITT population were similar for each treatment group (Table 1). All tumors were advanced and unresectable and all were histologically confirmed, with the exception of one patient in the Cilengitide and gemcitabine group. Three patients received other prior tumor-related therapies, and one patient in the gemcitabine only group received prior palliative radiotherapy. One patient in the Cilengitide and gemcitabine had a partial resection. The aforementioned 4 patients were granted waivers, because of protocol violations classified as minor and they were included in the PP population.

### Efficacy

**Figure 1:**
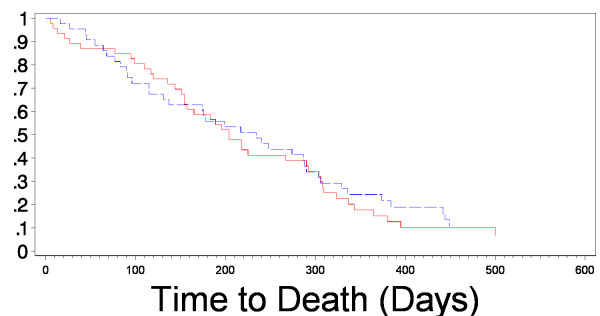
Time to death for the ITT population of patients randomized to receive (red) Cilengitide (600 mg/m^2^) and gemcitabine (1000 mg/m^2^) or (blue) gemcitabine alone (1000 mg/m^2^).

**Figure 2:**
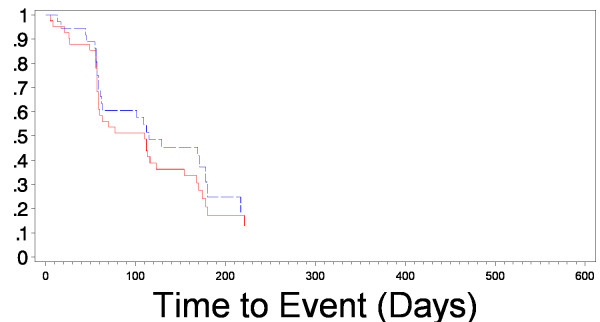
Progression free survival time for the ITT population of patients randomized to receive (red) Cilengitide (600 mg/m^2^) and gemcitabine (1000 mg/m^2^) or (blue) gemcitabine alone (1000 mg/m^2^).

Overall survival for each treatment group is presented in table 2 and figure 1 (progression free survival is shown in figure 2). There were no differences observed in survival between the ITT and PP populations. Although the initial treatment period was 6 months, patients responding to therapy could continue treatment for a further 6 months. At the time of the database closure, two patients with stable disease continued with the combination regimen on their own request because they had at least a subjective benefit from treatment. Response rate data are given in Table 3. There was no difference observed in tumor response between the ITT and PP populations.

### Biological response

The intra-individual course of CA 19-9 concentrations was observed to correlate with the clinical course, particularly in responders. The baseline levels were not well balanced in the treatment groups. (the median CA 19-9 concentration was greater in the gemcitabine group (1205.5 U/mL) than in the EMD 121974 and gemcitabine group (726.5 U/mL)). At week 5, CA 19-9 concentrations had declined in both groups and stayed relatively stable thereafter, remaining below the baseline concentration in both treatment groups at all subsequent time points.

No apparent relationship with plasma or serum VEGF, plasma or serum bFGF, or urinary bFGF concentrations was observed in either treatment groups, and there was no consistent pattern of change in any angiogenic growth factor level with time. The intra-individual course of these parameters was not observed to correlate with the clinical course. There were no apparent differences in the tumor marker data between the treatment groups at any time point. The results for the ITT and PP populations were broadly similar, except at week 17 in the PP population urinary bFGF was higher in the Cilengitide and gemcitabine group than in the gemcitabine alone group.

### Quality of Life

The number of patients receiving treatment at each time point decreased with time from 44 to 35 to 26 at weeks 1, 9 and 17 in the Cilengitide and gemcitabine group and from 42 to 30 to 20 in the gemcitabine alone group, respectively. At later time points the number of patients contributing to the QoL analysis was too small for any reliable conclusions to be made. A "moderate" difference was observed at week 9 for the pain scale in favor of gemcitabine alone whereas a "moderate" difference in favor of the combination treatment was seen in the insomnia scales at week 9 and in the diarrhea scale at week 17. The change from baseline was examined for the 15 subscales of the QLC-C30. In the Cilengitide and gemcitabine group, 61% and 56% of patients reported an improved global health status at weeks 9 and 17 compared with 50% and 47% in the gemcitabine alone treatment group. Patients who remained on the study for a longer period of time appeared to have a better QoL at baseline. The QLQ-PAN26 scale showed a "moderate" difference in favor of the gemcitabine alone group at week 9 for the flatulence scale, and at week 17 for the body image, weight, future health and planning activities scales. "Moderate" differences in favor of the combination treatment were observed at week 17 for altered bowel habit, health care satisfaction and sexuality scales.

### Drug exposure

Table 4 summarizes the drug exposure data. The mean number of completed cycles and the mean duration of treatment were slightly greater in the Cilengitide and gemcitabine treatment group (4.8 cycles and 134 days) than in the gemcitabine alone group (4.1 cycles and 120 days). The relative dose intensity (RDI) for both treatment groups was very close to the planned dose intensity and similar in both treatment groups.

### Safety

An overview of the adverse events is given in Table 5. All 86 patients experienced at least one adverse event during the treatment period of the study. The number of events was greater in the combination group than in the gemcitabine alone group. The most frequently reported adverse events that occurred during the treatment period were nausea (57% of patients), fatigue (49%), anemia (45%), vomiting (43%), abdominal pain (38%), dyspepsia (35%), thrombocytopenia (34%), and leukopenia (33%). Nausea (64% vs 50%), dyspepsia (41% vs 29%), dyspnea (27% vs 14%), shivering (21% vs 7%) and fever (34% vs 24%) were more frequent in the Cilengitide and gemcitabine group than the gemcitabine alone group, but taken solely the treatment-related events, only shivering remains more frequent (14% vs. 2%). Gamma-glutamyl transferase elevations (29% vs 16%), ascites (26% vs 9%), cholestasis (26% vs 7%), urinary tract infection (21% vs 5%) and jaundice (19% vs 2%) were reported more frequently in the gemcitabine alone group than in the Cilengitide and gemcitabine group. The incidence of other adverse events was observed to be similar in the treatment groups. A total number of 346 and 278 possibly related adverse events occurred in 84% (37) and 76% (32) of the patients in the Cilengitide and gemcitabine group and the gemcitabine alone group, respectively. The most frequently reported adverse events that were considered possibly related to the study medications were nausea, leukopenia, thrombocytopenia, and anemia (Table 6). All were of a comparable frequency in both groups, with the exception of leukopenia (36% vs 26%), which was reported by slightly more patients in the Cilengitide and gemcitabine group. In Cilengitide and gemcitabine group a number of 12 and in the gemcitabine alone group in total 8 possibly related serious adverse events were reported, respectively (Table 7).

The analysis of vital signs, physical examination, weight changes, chest X-rays, and ECGs did not raise any unexpected issues of clinical importance.

### Pharmacokinetic data

Eighteen patients were included in the pharmacokinetic sub-study (12 in the Cilengitide and gemcitabine group; and 6 in the gemcitabine alone group, data not shown). For Cilengitide non-compartmental pharmacokinetic analysis was performed on 11 data sets, 10 in week 2 and 1 in week 3. AUC_extra _provided more than 25% of AUC_0-∝ _for one patient, and so the value and derived parameters (AUC_0-∝_, λ_z_, CL, Vss) were flagged as unreliable and not included in the statistical analysis. Maximum plasma concentrations of Cilengitide were, in general, reached at 1 h post-dose (i.e. at the end of infusion). In most profiles, a distribution phase could be identified upon visual inspection. Thereafter, concentrations declined with a t_1/2 _of about 3 h and were thus measurable over the sampling period up to 12 h after start of infusion. There was no apparent difference between mean concentrations measured at the end of infusion in Week 1 (75410 ± 31530 ng/mL) in comparison to those in Week 2 (62182 ± 12345 ng/mL). The pharmacokinetic parameters of Cilengitide are summarized in Table 8.

For gemcitabine, maximum concentrations were generally attained at 0.5 h post dose (i.e. at the end of infusion). In accordance with the literature^34^, concentrations declined rapidly; at 2 h after start of infusion they were below the lower limit of quantification (LLQ) in about one third of the subjects and at 3 h after start of infusion nearly all concentrations were below LLQ. To compare maximum concentrations between treatment groups median instead of mean concentration values were used due to the comparatively high variability of single values. Medians of concentrations were comparable across the treatment groups. For the Cilengitide and gemcitabine group median concentrations were 6960 ng/mL (n = 12) and 7490 ng/mL (n = 11) in Weeks 1 and 2, respectively. For gemcitabine alone, median concentrations of 10600 ng/mL (n = 5) and 6060 ng/mL (n = 5) were observed for Weeks 1 and 2, respectively. Mean and median concentrations measured at 1 h and 2 h after start of infusion were of the same magnitude across all treatment groups.

## Discussion

This was a phase II, randomized, open-label study of gemcitabine with or without the cyclic pentapeptide Cilengitide conducted in chemotherapy-naive patients with unresectable pancreatic cancer. The patient demographic parameters, past medical history, and concomitant medications, were balanced across the treatment groups. The TNM classifications for the study population were comparable between treatment groups. All the patients had a clinical tumor stage at the start of the study of at least stage III (clinically unresectable), with the majority of patients being classified as stage IV.

### Efficacy

The median overall survival times of 7.7 months for patients receiving gemcitabine alone and 6.7 months for patients receiving Cilengitide and gemcitabine compare with the results from phase III trials, which have reported median survival times of 4.6 to 6.6 months [27-30] for gemcitabine alone. Both the response rate (17% vs. 14%) and the tumor growth control rates (54% vs. 56%) were similar in the combination treatment group and the gemcitabine alone group of patients. These overall response rates are in the previously reported range of 6% to 26% [31-34], which have been observed for single agent gemcitabine in patients with pancreatic cancer. Although there are currently few published studies for Cilengitide, in a phase I study in advanced cancer prolonged stable disease was seen in two patients with renal cell cancer and one patient with colorectal cancer [35]. Other recent approaches towards antiangiogenic or other targeted therapies in combination with gemcitabine like the treatment with the chimeric monoclonal EGFR-antibody cetuximab [30] or the anti-VEGF-agent bevacizumab [31] seem to be more promising regarding response rates (12,2 % and 21 % respectively) but not regarding median overall survival times (7.1 and 8,8 months respectively). Phase III trials with this agents are currently ongoing.

There were no apparent differences observed in the present study between treatment groups in the changes in serum marker levels over time. Despite the high variability of the CA19-9 data, the intra-individual course of CA19-9 concentrations went in line with the clinical course, particularly in responders, consistent with reports in the literature [36,37]. No apparent relationships were observed between the plasma or serum VEGF or bFGF, or urinary bFGF concentrations and either treatment group, nor was there any consistent pattern in the changes in the angiogenic growth factor levels with time. The evaluation of biological markers of angiogenesis was carried out to investigate their potential as surrogate markers of anti-tumor anti-angiogenic activity. The lack of change in the levels of the markers with time, also seen in phase I studies of Cilengitide [38], can be interpreted two ways. Either the finding is consistent with the lack of clinical benefit for Cilengitide or it suggests that the markers studied are not the best surrogate markers of the activity of an integrin inhibitor. Other approaches are being investigated such as dynamic contrast magnetic resonance imaging of tumor perfusion, the level of endothelial cell apoptosis and microvessel density as a measure for tumor angiogenesis, but the lack of clinical data for integrin antagonists [39-41] leaves no room for conclusions.

With regard to the QoL scores, from week 9 onwards the dropout rate was considerable, resulting in a potential overestimation of the functioning scales and underestimation of the symptom scores. However, a higher number of patients reported an improved QoL score in the Cilengitide and gemcitabine group compared with the gemcitabine alone treatment group. Although again drawing firm conclusions is difficult, the data do show that the combined treatment had no adverse effects on patient QoL compared with gemcitabine alone.

### Safety

The number of cycles received and the mean duration of treatment were broadly similar for the two treatment groups, and the RDIs were very close to the planned dose intensity. The profile of adverse events was consistent with the underlying disease and was similar across the treatment groups, with the exception of leukopenia (most frequent in the Cilengitide and gemcitabine group). Although the total number of adverse events was higher in the drug combination group, the patients in the Cilengitide and gemcitabine group were seen more frequently by the investigators than patients in the gemcitabine alone group, and together with the open-label nature of this study may have contributed to differences between the two treatment groups. Serious adverse events were reported more frequently by patients in the gemcitabine alone group (71%) than in the Cilengitide and gemcitabine group (55%), and might indicate that the combination treatment was reducing some of the symptoms of the disease. The lack of any Cilengitide exacerbation of gemcitabine toxicity is consistent with the findings from the published phase 1 study of single agent Cilengitide where no dose limiting toxicity was recorded and it was not possible to define a maximum tolerated dose [42].

Changes in biochemical, hematological, and urinalysis parameters during the study did not indicate any patterns for clinical concern and mainly reflected the progression of the underlying disease. There was no excess of increased elevations of alkaline phosphatase or bilirubin levels in the patients who received Cilengitide and gemcitabine in comparison with gemcitabine alone. No treatment-associated death were observed on study.

Vital signs, physical examinations, weights, chest X-rays, and electrocardiograms also did not show any matters of clinical concern. Overall, the safety evaluations suggest that Cilengitide did not adversely affect the safety and tolerability of gemcitabine in the group of patients studied.

### Pharmacokinetics

The mean Cilengitide CL was 54.2 ml/min/m^2^, which is low compared with hepatic and renal blood flow (and less than glomerular filtration rate) in man. Volume of distribution at steady state ranged between 8 and 16 L/m^2^, nominally equivalent to the extracellular fluid volume. The small volume of distribution and low plasma clearance manifests as a relatively short apparent terminal half-life of 3 hours. The short half-life of Cilengitide may have contributed to the lack of clinical benefit seen in this study. Continuous IV infusions or more frequent dosing schedule are therefore worth exploring in future studies. Pharmacokinetic parameters were similar to those from a published study of Cilengitide alone in patients with advanced solid tumors [43]. This finding indicates that the pharmacokinetics of Cilengitide are unaffected by the concomitant administration of gemcitabine. Gemcitabine concentrations were also in good agreement with the literature values [44] and comparable across the treatment groups suggesting the absence of a clinically relevant impact of Cilengitide on gemcitabine pharmacokinetics.

## Conclusion

The combination of Cilengitide and gemcitabine was well tolerated with no observed differences in the treatment groups on either adverse effects on the safety, tolerability and pharmacokinetics.

Efficacy results compare with previously reported gemcitabine single agent studies in this setting and considering that there was no inference planned, no difference was observed between the treatment groups of this trial.

## Authors' contributions

HF, AT, AK, and JN analyzed the data and drafted the manuscript. All other authors provided medical care, recruited patients and provided randomization. All authors read and approved the final version of the manuscript.

## Pre-publication history

The pre-publication history for this paper can be accessed here:



## Figures and Tables

**Table 1:** Patient Characteristics

		**Cilengitide + Gemcitabine n = 46**	**Gemcitabine n = 43**	**Total n = 89**
Age, years	Median	68	66	67
	Range	40–80	56–80	40–80
Gender (n, %)	Male	26 (57%)	18 (42%)	44 (49%)
	Female	20 (43%)	25 (58%)	45 (51%)
Stage	III	3 (7%)	4 (9%)	7 (8%)
	IV	3 (7%)	1 (2%)	4 (4%)
	IVa	3 (7%)	4 (9%)	7 (8%)
	IVb	37 (80%)	34 (79%)	71 (80%)
KP score at screening	70	3 (7%)	2 (5%)	5 (6%)
	80	19 (41%)	18 (42%)	37 (42%)
	90	18 (39%)	17 (40%)	35 (40%)
	100	6 (13%)	6 (14%)	12 (13%)

Abbreviation: KP, Karnofsky performance.

**Table 2:** Survival Data

	**Cilengitide + Gemcitabine n = 46**	**Gemcitabine n = 43**
Number of deaths	41	37
Median survival (95% CI), days	204 (154–292)	234 (137–290)
1-year survival (95% CI)	0.15 (0.04–0.26)	0.24 (0.11–0.37)
Median PFS (95% CI), days	110 (59–154)	115 (62–178)

Abbreviation: CI, confidence interval.

**Table 3:** Response Rate Data

	**Cilengitide + Gemcitabine n = 46**		**Gemcitabine n = 43**	
	
	No. patients	%	No. patients	%
Complete response	0	0%	1	2%
Partial response	8	17%	5	12%
Stable disease	17	37%	18	42%
Progressive disease	12	26%	10	23%
Missing/not assessable	9	20%	9	21%

Response rate	8	17%	6	14%
Tumor growth control	25	54%	24	56%

**Table 4:** Drug Exposure

	**Cilengitide + Gemcitabine n = 44**		**Gemcitabine n = 42**
	
	Cilengitide	Gemcitabine	Gemcitabine
Mean duration ± SD, days	134 ± 89		120 ± 75

Median duration (range), days	133 (1–344)		135 (1–330)
Mean no. cycles ± SD	4.8 ± 4.0		4.1 ± 2.7
Median no. cycles (range)	4.5 (0–19)		4.5 (1–12)
Mean RDI ± SD 1^st ^6 mths (range), %	93 ± 11	94 ± 11	89 ± 15
Median RDI 1^st ^6 mths (range), %	97 (53–101)	99 (55–104)	96 (47–105)

Abbreviations: SD, standard deviation; RDI, relative dose intensity; mths, months.

RDI = % of planned total dosage

**Table 5:** Overview of Adverse Events

**Event**	**Grade**	**Cilengitide + Gemcitabine n = 44**		**Gemcitabine n = 42**	
		
		No.	Pts (%)	No.	Pts (%)
AE	All	923	100%	755	100%
	Grade 1/2	812	27%	617	17%
	Grade 3/4	111	73%	138	83%

SAE	All	50	55%	64	71%

Adverse events with onset during the treatment phase.

Abbreviations: Pts, patients; AE, adverse event; SAE, serious adverse event.

**Table 6:** Adverse events occurring in at least 5% of patients considered to be possibly related to treatment

	**Adverse events (% of patients)**	
	**Cilengitide + Gemcitabine n = 44**	**Gemcitabine n = 42**
Nausea	36%	38%
Leukopenia	36%	26%
Thrombocytopenia	34%	33%
Anemia	27%	26%
Fatigue	23%	19%
Vomiting	18%	26%
Fever	11%	10%
Shivering	14%	2%
Peripheral edema	9%	5%
Headache	9%	0%
Sweating increased	11%	2%
Asthenia	7%	10%
Edema	7%	5%
Dyspepsia	7%	7%
Anorexia	7%	14%
Hair changes	5%	12%

**Table 7:** Serious adverse events possibly related to study treatment

**Treatment group**	**Adverse event**	**Grade**	**Number of events**
Cilengitide and gemcitabine	Myocardial infarction	4	1
	Apoplexia	4	1
	Thrombocytopenia	3	1
	Exanthema	1	1
	Weakness	3	2
	Nausea	3	1
	Anaphylactic reaction	4	1
	Pulmonary embolism	4	1
	Gastrointestinal hemorrhage	3	1
	Hypotension^1^	3	1
	Erysipelas^1^	3	1
Gemcitabine	Anorexia	3	1
	Exanthema	2	1
	Depression	3	1
	Dyspnea	3	2
	Epistaxis	2	1
	Pneumonia	2	1
	Condition reduced^1^	3	1

^1^Relationship "not assessable" by investigator; therefore, considered to be possibly related.

**Table 8:** Cilengitide Pharmacokinetic Data

**Parameter (unit)**	**n**	**Geomean**	**Min**	**Max**
C_max _(ng/mL)	11	61163	50100	85500
t_max _(h)	11	1*	1	1.25
AUC_0-t _(ng/mL*h)	11	182069	128234	334829
AUC_0-∝ _(ng/mL*h)	10	183968	134307	243990
λ_z _(1/h)	10	0.230	0.155	0.278
t_1/2 _(h)	10	3.0	2.5	4.5
CL (mL/min/m^2^)	10	54.2	41.0	72.5
V_ss _(mL/m^2^)	10	12100	8299	16384

Data are pharmacokinetic parameters of Cilengitide one hour after an intravenous infusion.

*Median is given.

Abbreviations are: n, number; Geomean, geometric mean; Min, minimum; Max, maximum; C_max_, the observed maximum plasma concentration; t_max_, time to reach C_max_; AUC, area under the curve; λ_z_, apparent first-order rate constant; t_1/2_, apparent terminal half-life associated with negative terminal slope; CL, total body clearance of drug from plasma; V_ss_, volume of distribution at steady state.
